# Novel multi-responsive P2VP-*block*-PNIPAAm block copolymers via nitroxide-mediated radical polymerization

**DOI:** 10.3762/bjoc.6.89

**Published:** 2010-08-20

**Authors:** Cathrin Corten, Katja Kretschmer, Dirk Kuckling

**Affiliations:** 1 Fachrichtung Chemie und Lebensmittelchemie, Technische Universität Dresden, D-01062 Dresden, Germany; 2 Department Chemie, Universität Paderborn, Warburger Str. 100, D-33098 Paderborn, Germany

**Keywords:** block copolymers, *N*-isopropylacrylamide, nitroxide-mediated radical polymerization, stimuli-responsive polymers, 2-vinylpyridine

## Abstract

Linear soluble multi-responsive block copolymers are able to form so called schizophrenic micelles in aqueous solution. Here, such polymers are prepared via nitroxide-mediated radical polymerization (NMRP). In a first step nitroxide-terminated poly(2-vinylpyridine) (P2VP) was prepared with different molecular weights and narrow molecular weight distributions. The best reaction conditions, optimized by kinetic studies, were bulk polymerization at 110 °C. Using P2VP as a macroinitiator, the synthesis of new soluble linear block copolymers of P2VP and poly(*N*-isopropylacrylamide) (PNIPAAm) (P2VP-*block*-PNIPAAm) was possible. The nitroxide terminated polymers were characterized by nuclear magnetic resonance (NMR) spectroscopy, size exclusion chromatography (SEC) and matrix-assisted laser desorption ionization time-of-flight mass spectrometry (MALDI-TOF MS). Thermal properties were investigated by the differential scanning calorimetry (DSC). Block copolymers showed pH- and temperature-responsive solubility in aqueous media. By increasing the P2VP content, the phase transition temperature shifted to lower temperatures (e.g. 26 °C for P2VP_114_-*block*-PNIPAAm_180_). Depending on the resulting block length, temperature and pH value of aqueous solution, the block copolymers form so called schizophrenic micelles. The hydrodynamic radius *R*_h_ of these micelles associated with pH values and temperature was analyzed by dynamic light scattering (DLS). Such kind of block copolymers has potential for many applications, such as controlled drug delivery systems.

## Introduction

Functional polymers have attracted much attention because of their technological and scientific importance. Polymers, which respond with large property changes to small external chemical or physical stimuli, are so called “smart”, “responsive” or “intelligent” polymers [1–2], constitute a very interesting group of functional polymers. Such polymers have found applications as reactive surfaces [3], in drug delivery and separation systems [4], as well as chemo-mechanical actuators [5], e.g., in valves where their characteristics have been studied extensively by a large range of methods [6–7].

One of the most intensively studied polymers in this field is poly(*N*-isopropylacrylamide) (PNIPAAm), which exhibits a sharp phase transition in water at 32 °C [8]. PNIPAAm undergoes a temperature-induced collapse from an extended coil to a globular structure, a transition revealed on the macroscopic scale by sudden decrease in the solubility of PNIPAAm. This behavior is derived from changes in the balance of interactions between hydrophilic and hydrophobic groups in the polymer chains at the critical temperature.

In order to prepare multi-responsive polymers, it is necessary to combine different kinds of monomers. For this purpose the preparation of defined block copolymers with different architectures is demanded. Amphiphilic or smart block and graft copolymers are already known in the literature [9]. Block copolymers in a wide range of variety are synthesized by using living anionic polymerization [10], living cationic polymerization [11] or controlled radical polymerizations techniques [12]. The development of the controlled radical polymerization (CRP), based on the idea of reversible chain termination, decreases the disadvantage of the free radical polymerization and permits the synthesis of defined block copolymer structures [13]. The growing demand for well-defined and functional soft materials in nanoscale applications has led to a dramatic increase in the development of procedures that combine architectural control with flexibility in the incorporation of functional groups. Thus, there has been a considerable increase in the understanding of a variety of controlled polymerization strategies [14–17] over the last few years. This includes nitroxide-mediated radical polymerization (NMRP) [18], atom transfer radical polymerization (ATRP) [19–20] and radical addition fragmentation chain transfer procedures (RAFT) [21–22]. The controlled polymerization of styrene, and analogous monomers such as 2-vinylpyridine (2VP), is one point of interest because at pH values lower than 5 it is possible to protonate the 2VP units and hence P2VP can be used as a pH-responsive component. Several techniques such as NMRP, ATRP and RAFT led to well-defined homo and block copolymers of different architectures whose behavior was investigated in solution and on surfaces [23–24].

The synthesis of NIPAAm homopolymers through different controlled polymerization techniques is described in the literature. Using RAFT it was possible to obtain amphiphilic block copolymers of PNIPAAm (hydrophilic) and poly(styrene) (PS) or poly(*tert*-butylmethacrylate) (PtBMA) as the hydrophobic compounds [25]. The design of bi-responsive narrowly distributed block copolymers consisting of NIPAAm and acrylic acid (AAc) was also feasible [26]. By the use of the ATRP catalyst system of tris(2-dimethylaminoethyl)amine (Me_6_TREN) and Cu(I) chloride, well-defined PNIPAAm could be synthesized at room temperature [27]. Several graft copolymers are described in previous reports such as Chitosan-*graft*-PNIPAAm [28] and PNIPAAm-*graft*-P2VP polymers [29]. Both polymers show a temperature- and pH-responsive phase behavior in aqueous solutions.

**Scheme 1:**
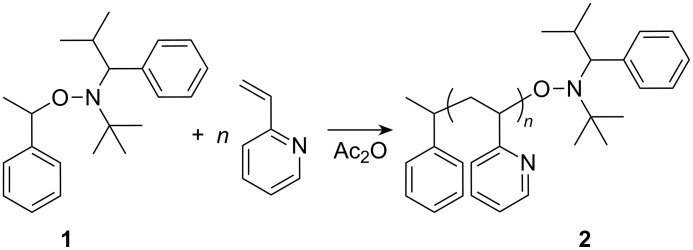
Synthesis of the nitroxide-terminated P2VP-macroinitiator.

While there are advantages and disadvantages to each procedure, our recent work concentrated on nitroxide mediated processes because of the ease of the reaction and the absence of transition metal impurities (binding easily to 2VP moieties) in the product. A major recent advance in nitroxide mediated polymerization has been the development of a hydrido nitroxide, in which the presence of a hydrogen atom on the α-carbon leads to a significant increase in the range of vinyl monomers that undergo controlled polymerization [30]. From that point of view, alkoxyamine **1** as an initiator for the polymerization of the 2VP has been selected and the resulting polymer was used as a macroinitiator **2** (Scheme 1).

Amphiphilic diblock copolymers undergo a self-assembly micellar process in solvents that are selective for one of the blocks [31]. By choosing selective conditions for each block, conventional micelles and so-called inverse micelles can be formed. In recent papers some examples of so called schizophrenic micelles are described [31–32]. In this case hydrophilic AB diblock copolymers can form micelles in aqueous solution, in which the A block forms the inner core and inverted micelles (with the B block forming the inner core) [33]. Armes et al. described the synthesis of a diblock copolymer with two weak polybases (poly(2-(*N*-morpholino)ethyl methacrylate-*block*-2-(diethylamino)ethyl methacrylate) PMEMA-*block*-PDEA) via group transfer polymerization. By adjusting the pH value of the solution, it was possible to from stable micelles with PDEA cores. The formation of inverted micelles (PMEMA core) was achieved by a “salting out” effect by adding electrolytes to the aqueous solution.

The synthesis of polyampholytes from P2VP as a basic block was reported in several papers, e.g., poly(2-vinylpyridine-*block*-sodium-4-styrenesulfonate) [34], poly(2-vinylpyridine-*block*-acrylic acid) [35], and poly(2-vinylpyridine-*block*-ethylene oxide) [31]. In this case according to the corresponding pH value of the solution, it was possible to obtain precipitation, aggregation or micellation.

An example of double-responsive diblock copolymers is reported by Müller et al. [26]. Diblock copolymers of poly(*N*-isopropylacrylamide-*block*-acrylic acid) were synthesized via RAFT. The resulting behavior in aqueous solution is influenced by hydrogen bonding interactions between the *N*-isopropylacrylamide and acrylic acid units.

Herein, we describe the synthesis of new multi-responsible diblock copolymers poly(2-vinylpyridine-*block*-*N*-isopropylacrylamide), which form schizophrenic micelles. Such micellation behavior is interesting for drug delivery systems in the gastro-intestinal tract [36–37].

## Results and Discussion

### Polymerization of 2-vinylpyridine

By using the unimolecular initiator 2,2,5-trimethyl-3-(1-phenylethoxy)-4-phenyl-3-azahexane (St-TIPNO) (**1**), it was possible to synthesize macroinitiators based on 2VP. In order to analyze the controlled character of the 2VP homo polymerization (Scheme 1), a kinetic study of this reaction with varying synthesis parameters (temperature, time and different molar ratios of [initiator]/[monomer]) was performed. A constant value of 2 equiv Ac_2_O according to the amount of the alkoxyamine to each reaction mixture was added. The necessary addition of acetic anhydride or other organic acids is described in the literature [38].

After starting the reaction of 2VP in bulk at different temperatures (90 °C, 110 °C, 130 °C), a sample of 0.2 mL of the reaction mixture was taken after certain periods of time. 0.1 mL of this portion was analyzed by ^1^H NMR spectroscopy in perdeuterated chloroform. The conversion was calculated by using the typical signal for CH=CH_2_ of the monomer at 5.45 ppm and the peak at 8.44 ppm for the CH_arom_–N of the 2VP polymer. The molecular weight and molecular weight distribution were determined by SEC measurements using THF as the mobile phase.

**Figure 1:**
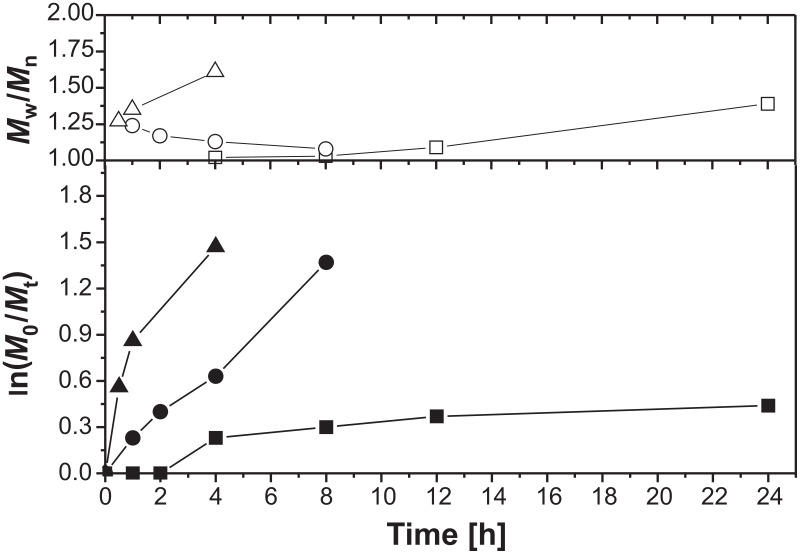
Plots of ln(*M*_0_/*M*_t_) and molecular weight distribution vs time of the homopolymerization of 2VP at ■ 90 °C, ● 110 °C, ▲ 130 °C (molar ratio [initiator]/[monomer] 1:140).

Figure 1 shows the plots of ln(*M*_0_/*M*_t_) and molecular weight distribution versus time at different temperatures. Here, characteristics known for controlled polymerizations are found, i.e., conversion increases within prolonged reaction time, molecular weight increases linearly with conversion, and products possess narrow molecular weight distribution. Increased temperature caused an enhancement of the reaction speed, which was also influenced by the molar ratio of [initiator]/[monomer]. This corresponds to various reports on the existence of the persistent radical effect (PRE) as a kinetic phenomenon [39].

**Figure 2:**
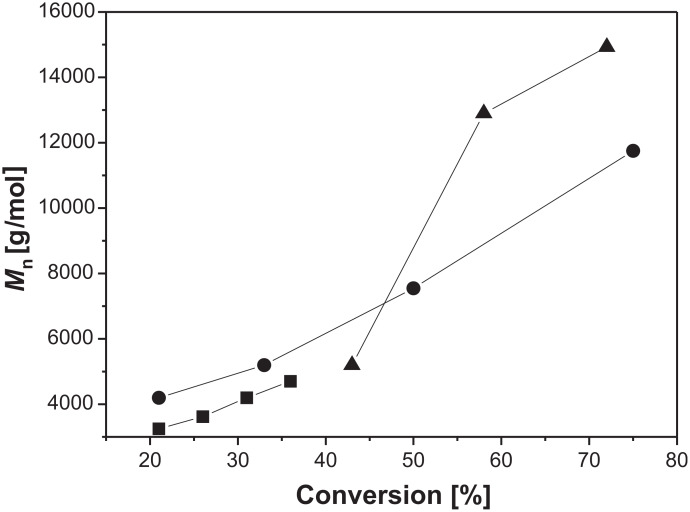
Plots of number average molecular weight vs conversion for the homopolymerization of 2VP for ■ 90 °C, ● 110 °C, ▲ 130 °C, (molar ratio [initiator]/[monomer] 1:140).

At 90 °C a very long induction period was found. After 4 h a conversion of 21% and after 8 h of 36% was determined. Despite this low polymer conversion, the molecular weight distribution was very narrow. However, for a practical process this reaction temperature is not useful because at extended reaction times, side reactions, e.g., elimination of the end capping nitroxide group by β-elimination, can occur terminating chain growth. Hence, an increased molecular weight distribution was observed. At 130 °C the reaction was very fast leading to a strong increase in conversion. After 30 min 40% of polymer was obtained. Apart from a high conversion, a broad molecular weight distribution of the products was obtained. The best reaction temperature was found to be 110 °C. At this temperature a linear relationship between conversion and reaction time was observed. For example, after 6 h a conversion of 50% corresponding to a molecular weight of 7550 g/mol (Figure 2).

**Figure 3:**
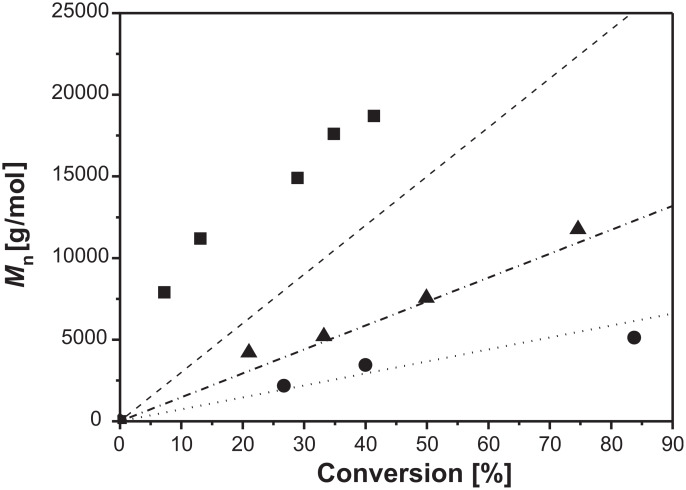
Plot of number average molecular weight vs conversion for the homopolymerization of 2VP at 110 °C with different molar ratios of [initiator]/[monomer] ■ 1:210, ▲ 1:140, ● 1:70. The dashed lines describe the theoretical behavior.

During the progress of the reaction, a decrease of the molecular weight distribution could be found corresponding to the living character of this reaction [14]. In Figure 3 the molecular weight of P2VP prepared with different molar ratios of [initiator]/[monomer] at a constant temperature of 110 °C, compared with the theoretical molecular weights as function of conversion, are presented. The ratios [initiator]/[monomer] of 1:70 and 1:140 showed a linear increase of the molecular weight with increasing conversion, and are in good agreement with the calculated data. For a [initiator]/[monomer] ratio of 1:210, the obtained molecular weights are higher than the calculated ones. This is an indication that the amount of initiator was too small to control the polymerization. An increasing number of non-living processes occurred, yielding polymer chains having higher molecular weights.

**Figure 4:**
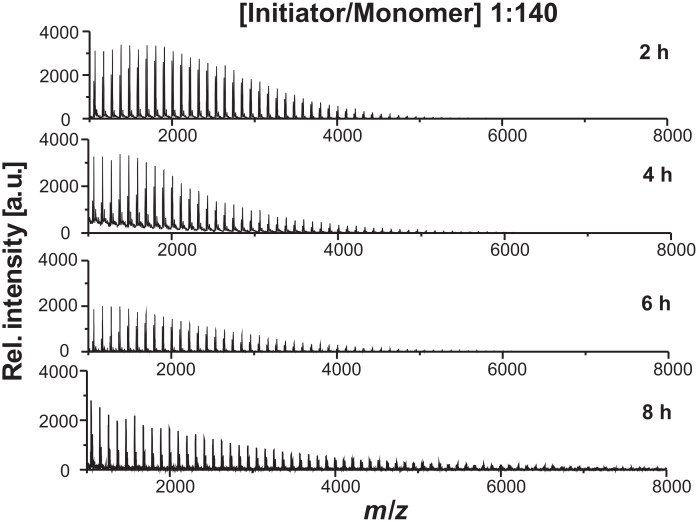
MALDI-TOF MS of P2VP obtained for polymerizations stopped after 2, 4, 6, and 8 h. The samples were prepared by the dried droplet method dissolving the polymer, DT, and KOTf in THF. To gain representative information, the spot was probed at several locations and 100 spectra were accumulated.

By performing NMRP on 2VP, the resulting polymer should possess defined end groups (Scheme 1). In order to analyze the polymer structure MALDI-TOF MS was employed. The spectra of samples obtained from the polymerization at 110 °C with a [initiator/monomer] ratio of 1:140 stopped after 2, 4, 6 and 8 h are depicted in Figure 4. All distributions of the polymers exhibited differences between the *m/z*-peaks in the MALDI-TOF spectra that can be attributed to the weight of the 2VP monomer unit. In contrast to the molecular weight data obtained by SEC analysis, the molecular weight determined by MALDI-TOF MS only increased from *M*_n_ = 1530 g/mol to *M*_n_ = 2800 g/mol. One reason might be the laser energy used to desorb the polymer led to P2VP chain degradation or fragmentation. Since SEC calibration has been done with P2VP standards, one can assume that the different results can be ascribed to significant ionization biases during MALDI-TOF analysis leading to incorrect molecular weights.

**Scheme 2:**
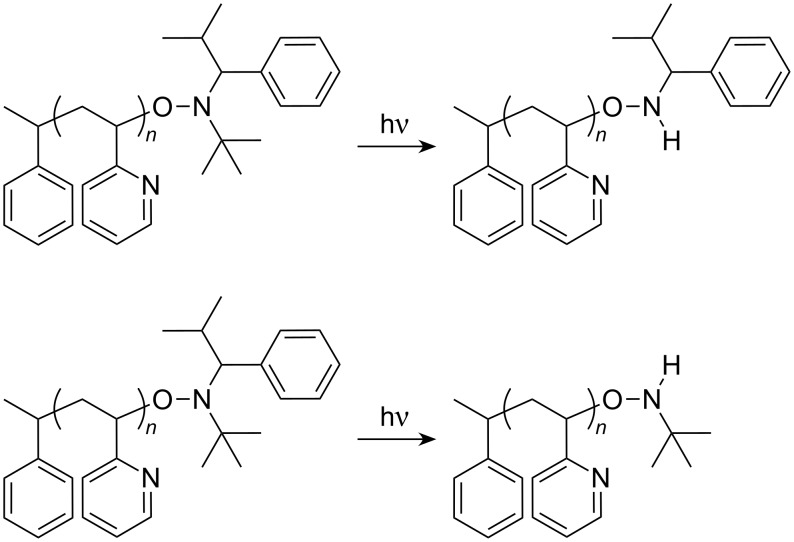
Degradation of nitroxide-terminated P2VP-macroinitiator by laser light irradiation.

In addition, Dempwolf et al. [40] tested different alkoxyamines in different MALDI experiments. Supported by a comparison with other methods, they postulate a fragmentation mechanism inside the nitroxide-group, which takes place during the MALDI measurement. Disregarding the mechanism, Scheme 2 describes the possible reactions.

**Figure 5:**
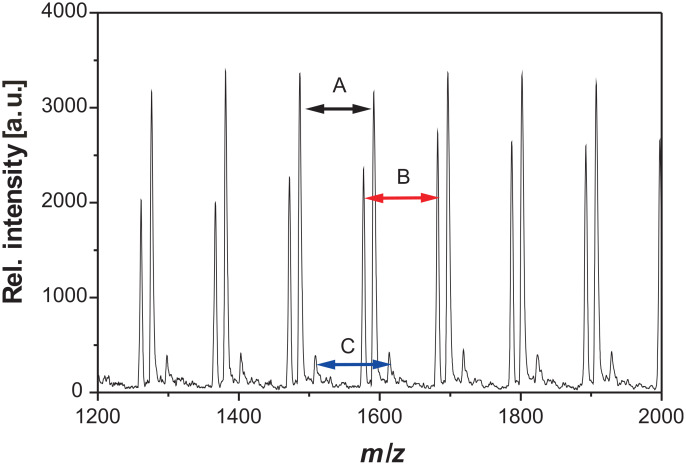
MALDI-TOF MS of P2VP obtained for polymerizations stopped after 2 h. The samples were prepared by the dried droplet method dissolving the polymer, DT, and KOTf in THF.

Since this process is accompanied by other degradation processes such as ß-abstraction [41] and by the instability of the C–O bond, it was not possible to detect the complete end groups. Figure 5 shows a section of typical spectra. Three different distributions could be observed, each of them has a repeating unit of *m/z* = 105.13, which corresponds to the monomer unit of 2VP. In Table 1 possible polymer structures according to suitable sum formula are summarized. For peak B different compositions could be assigned. At this point it is not possible to decide, if the measured distribution belongs to a thermal or nitroxide started polymer chain.

**Table 1:** End group determination of the macroinitiators via MALDI-TOF MS.

Peak	*m*/*z* [exp.]	*m*/*z* [th.]	Chain	*n*	Cation	Δ *m*/*z*

A	1591.12	1597.97	[CH_3_(C_7_H_6_)–[2VP]*_n_*–C_4_H_10_NO]	13	K^+^	6.82
B	1577.12	1578.08	[CH_3_(C_7_H_6_)–[2VP]*_n_*–H]	14	H^+^	0.96
		1579.07	[CH_3_(C_5_H_6_N)–[2VP]*_n_*–H]	14	H^+^	1.95
C	1513.13	1510.90	[CH_3_(C_7_H_6_)–[2VP]*_n_*–H]	13	K^+^	2.23

In summary, the analysis of such nitroxide capped polymers by MALDI-TOF MS is complex. However, by using St-TIPNO as an alkoxyamine initiator, it was possible to obtain 2VP-macromonomers of different molecular weights with narrow molecular weight distributions.

### Synthesis of linear multi-responsive soluble polymers

**Scheme 3:**
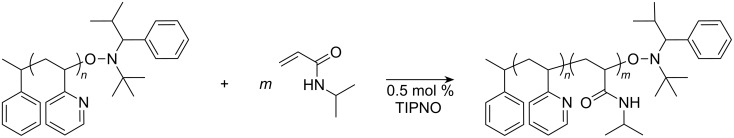
Synthesis of the bi-responsive block copolymers.

**Figure 6:**
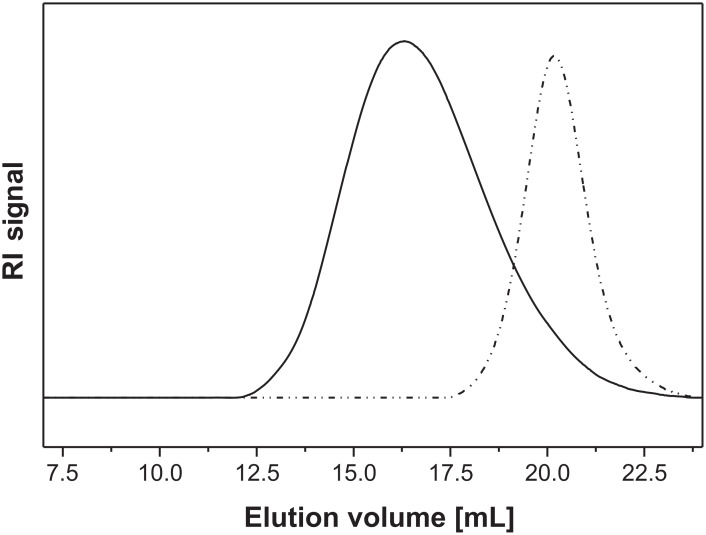
SEC traces for P2VP-*block*-PNIPAAm (solid line) and P2VP macroinitiator (dashed line).

Based on the results of homo polymerization for NIPAAm known from literature [9], it was possible to create suitable block copolymers with nitroxide-terminated P2VP macroinitiators and NIPAAm (Scheme 3). Chain extension of nitroxide capped polymers is only possible in intact polymers. Figure 6 illustrates typical SEC traces for the NIPAAm containing block copolymers. The shift of the peak to a smaller elution volume relative to the macroinitiator indicated successful block copolymer formation. No shoulder or second peak at elution volumes for macroinitiator was found, indicating that most of the polymers possessed intact structure. Additionally, the SEC traces show an overlap between the two traces, which might be taken as hint for the existence of unreacted macroinitiator.

After 48 h at 135 °C block copolymers with an average yield of 65% could be obtained. As described in previous papers, the process of the NIPAAm polymerization with nitroxide-mediated compounds is neither a well-controlled process nor does it result in a real living character [41]. However, PNIPAAm homo polymer of *M*_n_ = 7500 g/mol could be obtained with a molecular weight distribution of 1.21. After block copolymerization, the molecular weight distribution increased. Nevertheless, for most of the block copolymers the molecular weight distribution remained moderate. The results of copolymer characterization are summarized in Table 2. DSC measurements revealed two separated *T*_g_s, indicating a microphase separation of the block copolymers in the dry state.

**Table 2:** Characterization of soluble linear P2VP-*block*-PNIPAAm copolymers created by NMRP.

Polymer	*M*_n_ [g/mol]	*M*_w_ / *M*_n_	*T*_c_ [°C]	*T*_g_ [°C]

PNIPAAm	7500	1.21	32.3	—
P(2VP)_22_-*block*-P(NIPAAm)_181_	16600	1.35	30.5	131.7
P(2VP)_85_-*block*-P(NIPAAm)_351_	22600	1.74	29.3	107.4 / 132.7
P(2VP)_105_-*block*-P(NIPAAm)_332_	28900	1.62	28.0	97.8 / 130.4
P(2VP)_114_-*block*-P(NIPAAm)_180_	21700	1.43	26.3	103.9 / 131.2
P(2VP)_114_-*block*-P(NIPAAm)_244_	24700	1.57	27.6	97.4 / 131.6
P(2VP)_114_-*block*-P(NIPAAm)_648_	45500	2.19	28.6	104.9 / 128.9

**Figure 7:**
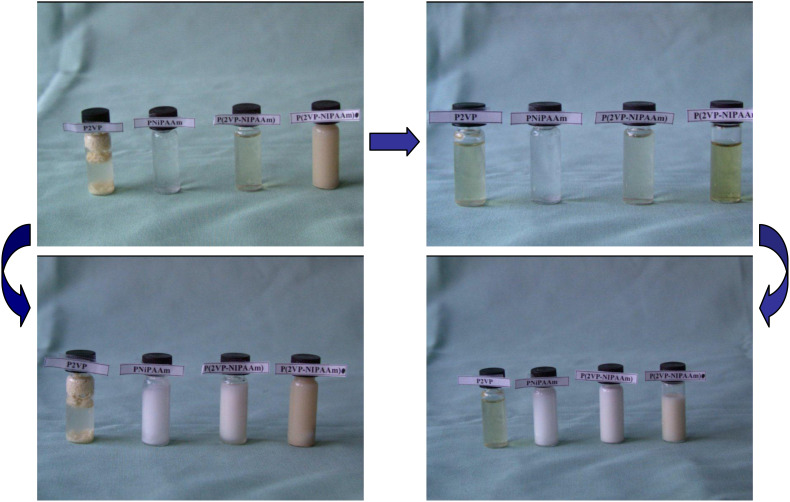
Demonstration of the solution behavior. Polymers from left to right: P2VP, PNIPAAm, P(2VP)_85_-*block*-P(NIPAAm)_351_, P(2VP)_114_-*block*-P(NIPAAm)_244_; a) pH7 and RT, b) pH 4 and RT, c) pH 7 and *T* > 35 °C, d) pH 4 and *T* > 35 °C.

Aqueous solutions of these block copolymers showed an LCST behavior. Due to the hydrophobic character of P2VP, the resulting polymers possessed lower phase transition temperatures compared to pure PNIPAAm [42]. With increase of the 2VP/NIPAAm ratio within the block copolymers, the critical temperature dropped to 26.3 °C. Although all polymers showed a temperature-dependent solution behavior, only block copolymers with a high P2VP content showed pH sensitivity. Solubilization of such polymers was possible below pH 5 only due to the protonation of pyridine moieties. In Figure 7 the solution behavior of such polymers is demonstrated.

Figure 7a shows that the P2VP macroinitiator and P2VP_114_-*block*-PNIPAAm_244_ were not soluble in aqueous solution of pH 7 at lower temperatures, while PNIPAAm and P2VP_85_-*block*-PNIPAAm_351_ were completely dissolved under these conditions. By increasing the temperature above 35 °C, none of polymers were soluble. A decrease of the pH value to pH 4, resulted in protonation of the P2VP fraction, which also led to completely soluble polymers at lower temperatures. The phase separation behavior was also observable at higher temperatures. Above the critical temperatures, all polymer solutions with a PNIPAAm fraction became opaque.

**Figure 8:**
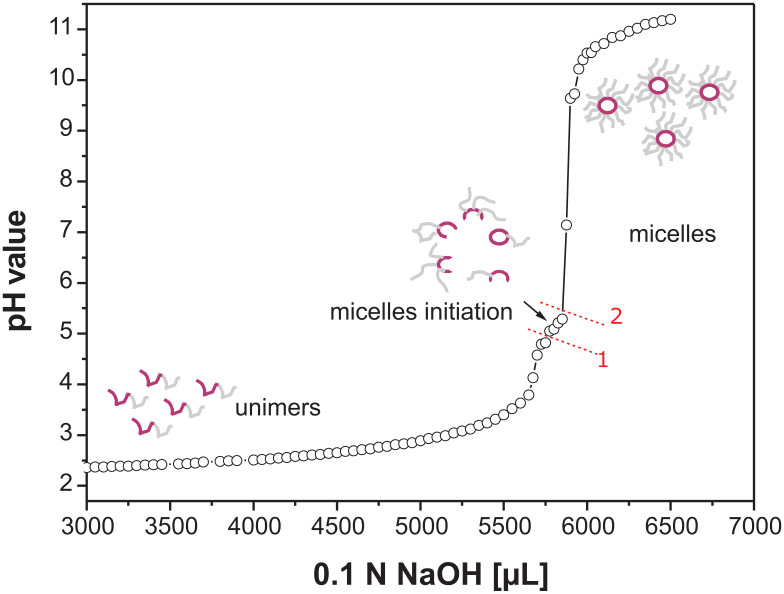
Titration of P2VP_105_-*block*-PNIPAAm_332_ (1g/L) in 0.02 N HCl with 0.1 N NaOH at room temperature.

A typical titration curve for the multi-responsive *block* copolymers is presented in Figure 8. By adding 0.1 N NaOH to a stirred solution of P2VP_105_-*block*-PNIPAAm_332_ in 0.02 N HCl, scattering polymer particles were produced at a pH range of 4–5 around the added NaOH droplets (high local concentration). When the solution is homogenized by stirring, the scattering disappeared. This indicates that the micelle formation is a dynamic and reversible process. When the pH value reaches 4.8 (point 1), aggregates were visible over the entire volume, and above 5.3 (point 2) the micelles formation was complete.

The p*K*_a_ for 2-ethylpyridine is 5.9. As described in the literature [31], due to the concentrated pyridine groups along the polymer backbone, the effective p*K*_a_ is lower than for this model substance as a result of charge repulsion along the chain, decreasing the p*K*_a_ value to 4.4. It has been shown [43] that the effective p*K*_a_ varies with the fraction of protonation of P2VP. Hence, by titration it is not possible to measure the real p*K*_a_.

**Figure 9:**
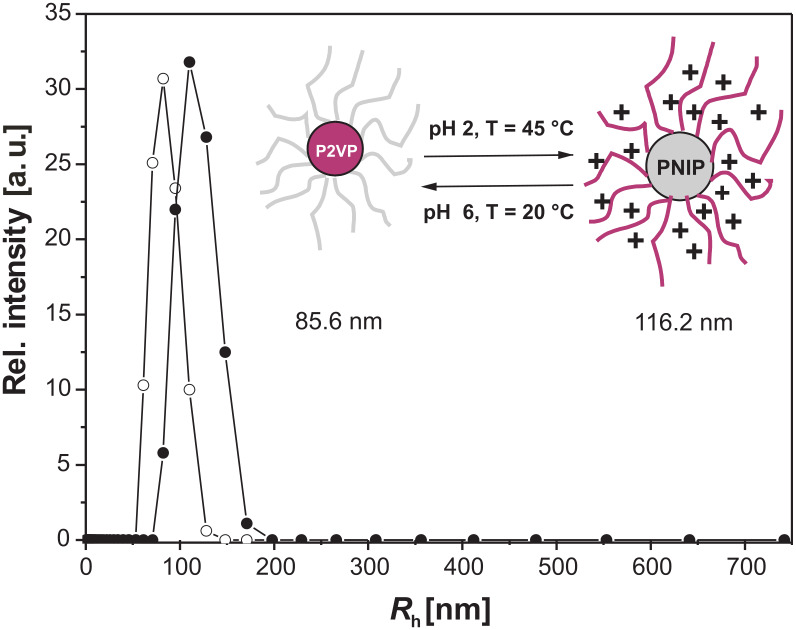
Hydrodynamic radius distribution of P(2VP)_105_-b-P(NIPAAm)_332_ at ○ pH 7, *T* = 20 °C and ● pH 2, *T*= 45 °C.

In order to investigate the size of the micelles, dynamic light scattering experiments were performed on diluted block copolymer solutions under various conditions. The resulting hydrodynamic radii of the diblock copolymers are summarized in Table 3. Polymers with short P2VP blocks, P2VP_85_-*block*-PNIPAAm_351_ and P2VP_22_-*block*-PNIPAAm_181_, behave similar to homo PNIPAAm at 20 °C. No association, due to the incorporation of the 2VP block, could be observed. The two aqueous solutions were completely clear and showed no scattering indicating that the polymers were molecularly dissolved. Above the critical temperature, micelles, with *R*_h_ of 55 nm and 79 nm, respectively, were formed stabilized by partly ionized P2VP blocks. Interestingly, the decrease of the pH value to pH 2 led to formation of large aggregates instead of micellation at higher temperatures. This instability of the polymeric material in the presence of an HCl solution above the critical temperature can be explained by increased ionic strength at pH 2. Chloride ions can bind to polar amide groups of the PNIPAAm units, and might interact with the water molecules associated with polar or hydrophobic polymer segments [44]. Hence, driving forces for inter- and intramolecular hydrophobic interactions are increased leading to a decrease in the stability of the NIPAAm polymers, which then tend to form larger aggregates. The protonated P2VP units are too small to inhibit this process. The results of the P2VP-*block*-PNIPAAm copolymers with longer P2VP segments showed the expected results. In neutral aqueous solutions, micelles with a hydrophobic P2VP core and an outer shell of PNIPAAm were formed. By increasing the temperature to 45 °C, the PNIPAAm units became more hydrophobic, and were not able to stabilize the micelles anymore. Finally, the micelles were forming large aggregates and precipitated. By dissolving the polymers in 0.02 N HCl at 20 °C, the P2VP segments were completely protonated forming soluble unimers. Polymers with larger P2VP/PNIPAAm ratios were forming inverted micelles above the critical temperature. The protonation of the 2VP units led to electrostatic repulsion and the longer P2VP blocks were able to stabilize the micelles preventing PNIPAAm forming larger aggregates even in diluted HCl. Due to the longer PNIPAAm block with respect to the P2VP block, micelles formed by a PNIPAAm core and by a P2VP outer shell showed larger hydrodynamic radius. The schizophrenic behavior of P2VP_105_-*block*-PNIPAAm_332_ is summarized in Figure 9.

**Table 3:** Dynamic light scattering characterization of bi-responsive P2VP-*block*-PNIPAAm copolymers in aqueous solution.

Polymer	Ratio P2VP/PNIPAAm	*R*_h_ pH 7 20 °C [nm]	*R*_h_ pH 7 45 °C [nm]	*R*_h_ pH 2 20 °C [nm]	*R*_h_ pH 2 45 °C [nm]

P(2VP)_22_-*block*-P(NIPAAm)_181_	1:8	4.5	55.3	5	Agg^a^
P(2VP)_85_-*block*-P(NIPAAm)_351_	1:4	8	79.4	7.5	Agg
P(2VP)_105_-*block*-P(NIPAAm)_332_	1:3	85.6	Agg	5	116.2
P(2VP)_114_-*block*-P(NIPAAm)_244_	1:2	71.2	Agg	6	112.3

^a^Agg: aggregates larger than *R*_h_ = 1100 nm.

## Conclusion

Well-defined P2VP macroinitiators were prepared using NMRP. The kinetic study showed the controlled behavior of the polymerization for this vinyl monomer. Best polymerizations were carried out in bulk at 110 °C with a molar ratio of [initiator]/[monomer] of 1:140. Under these conditions, nitroxide-terminated P2VP with different molecular weights and a narrow molecular weight distribution could be synthesized. It is well known that the MALDI process causes severe degradation of nitroxide end capped polymers. This fragmentation could be observed as well for P2VP. Despite this fact, chain extension of such polymers is only possible by intact polymers. No shoulder or second peak at elution volumes for macroinitiator was found indicating that most of the polymers possessed intact structure. Using P2VP as a macroinitiator, new soluble linear block copolymers of P2VP and PNIPAAm were synthesized, which showed a pH- and temperature-responsive solubility. With increased P2VP content, the phase transition temperature shifted to lower temperatures (e.g., 26 °C for P2VP_114_-*block*-PNIPAAm_180_). DLS measurements of the block copolymers underlined the multi-responsive and schizophrenic behavior in aqueous solutions. DSC measurement of the glass transition temperature revealed a microphase separation behavior for these block copolymers in the dry state.

## Experimental Section

### Materials

*N*-isopropylacrylamide (NIPAAm, Acros) was purified by recrystallization from hexane and dried in vacuum. 2-Vinylpyridine (2VP, 98 %, Merck) was stirred over calcium hydride for 24 h and freshly distilled before use. Dimethylformamide (DMF) was distilled over calcium hydride. All other chemicals were used as received.

### Characterization

NMR spectra were recorded on a Bruker NMR spectrometer DRX500. Elemental analysis was done with a Hekatech EA 3000 Euro Vector CHNSO Elementaranalysator. DSC measurements were carried out with a Mettler-Toledo DSC 30 to determine the glass transition temperature (*T*_g_) of the block copolymers (heating rate 10 °C/min) and with a TA Instruments DSC 2290 to measure the phase transition temperature (*T*_c_) (heating rate of 5 °C/min) as an average of 4 cycles. The polymer concentration was 50 mg/mL in a pH 4 buffer solution (CertiPUR^®^ Merck). Molecular weight and the molecular weight distribution of P2VP were determined by size exclusion chromatography with a JASCO instrument set up with UV and RI detector using a P2VP-calibration. The samples were measured at 30 °C in THF as the mobile phase with a flow rate 1 mL/min. BHT was used as an internal standard on Polymer Laboratories linear columns (PLgel MIXED-BLS 10 mm). The parameters of the copolymers were determined by size exclusion chromatography (SEC) with a PL120 instrument equipped with RI detector using PSS ‘GRAM’ columns using a P2VP-calibration. The samples were measured at 50 °C in dimethylacetamide (DMAc) containing 0.42 g/L lithium bromide as mobile phase with a flow rate of 1 mL/min. Matrix assisted laser desorption ionization time of flight mass spectrometry (MALDI-TOF MS) was performed on a BiFlex IV (Bruker Daltonics). 1,8,9-Anthracenetriol (DT) (Bruker Daltonics) was used as the matrix and potassium triflouromethanesulfonate (98% ACROS) was added to improve the ionization process. The samples were prepared by mixing THF solutions of the polymer, matrix and salt (10 mg/mL) in a typical ratio of 1:10:1 (v/v/v, polymer/matrix/salt), and a droplet (1 µL) of the mixture was dried on the target. As calibration standard poly(ethylene oxide) [*M*_w_ = 2000 g/mol, Sigma-Aldrich] was used.

Dynamic light scattering (DLS) was measured on a Zetasizer Nanoseries Nano-ZS (Malvern instruments) with a laser at 633 nm, a constant angle of 173° and a temperature of 25 °C. The hydrodynamic radius (*R*_h_) was calculated using the Stokes–Einstein relation. The polymeric solutions were prepared from double-destilled water or 0.02 M HCl (aq) solution with polymer concentration of 0.5 g/L. All solutions were prepared 60 min before measurements. The solutions were treated with ultrasound for 5 min and filtered through PES filters (pore size 0.45 µm).

### Synthesis

2,2,5-Trimethyl-3-(1-phenylethoxy)-4-phenyl-3-azahexane (St-TIPNO) (**1**) and the corresponding nitroxide 2,2,5-trimethyl-4-phenyl-3-azahexane 3-nitroxide (TIPNO) were prepared according to the literature [45–46].

### General procedure of 2VP polymerization

A mixture of 2VP, 0.1 mL acetic anhydride and the alkoxyamine **1** was degassed by three freeze/thaw cycles, sealed under argon, and heated at 110 °C for different periods of time. Afterwards the polymerization was stopped by cooling with liquid nitrogen. The reaction mixture was then diluted with THF and precipitated in *n*-pentane (ratio 1:5). The obtained powder was dried in vacuum to give the desired alkoxyamine-terminated P2VP.

### Preparation of multi-responsive block copolymers

A mixture of the alkoxyamine-terminated P2VP macroinitiator, NIPAAm, and TIPNO dissolved in DMF was degassed by three freeze/thaw cycles, sealed under argon and heated to 135 °C for 48 h. Afterwards the reaction was stopped by cooling with liquid nitrogen. The solvent was almost removed by evaporation under reduced pressure. The residue was redissolved in chloroform and precipitated in cold diethyl ether. The resulting brownish powder was dried in vacuum. Block copolymer was obtained with up to a yield of 65%.
